# CRISPR-mediated germline mutagenesis for genetic sterilization of *Anopheles gambiae* males

**DOI:** 10.1038/s41598-024-54498-8

**Published:** 2024-02-19

**Authors:** Andrea L. Smidler, Eryney Marrogi, Jamie Kauffman, Douglas G. Paton, Kathleen A. Westervelt, George M. Church, Kevin M. Esvelt, W. Robert Shaw, Flaminia Catteruccia

**Affiliations:** 1 Department of Immunology and Infectious Diseases, Harvard T.H. Chan School of Public Health, Boston, MA 02115 USA; 2 Department of Genetics, Harvard Medical School, Boston, MA 02115 USA; 3 Media Lab, Massachusetts Institute of Technology, Cambridge, MA 02139 USA; 4 Howard Hughes Medical Institute, Chevy Chase, MD 20815 USA; 5 Present Address: Department of Biology, University of California - San Diego, San Diego, CA 92093 USA; 6 Present Address: Department of Infectious Diseases, University of Georgia, Athens, GA 30602 USA

**Keywords:** Malaria, Genetic engineering, Entomology

## Abstract

Rapid spread of insecticide resistance among anopheline mosquitoes threatens malaria elimination efforts, necessitating development of alternative vector control technologies. Sterile insect technique (SIT) has been successfully implemented in multiple insect pests to suppress field populations by the release of large numbers of sterile males, yet it has proven difficult to adapt to *Anopheles* vectors. Here we outline adaptation of a CRISPR-based genetic sterilization system to selectively ablate male sperm cells in the malaria mosquito *Anopheles gambiae*. We achieve robust mosaic biallelic mutagenesis of z*ero population growth* (*zpg*, a gene essential for differentiation of germ cells) in F1 individuals after intercrossing a germline-expressing Cas9 transgenic line to a line expressing *zpg*-targeting gRNAs. Approximately 95% of mutagenized males display complete genetic sterilization, and cause similarly high levels of infertility in their female mates. Using a fluorescence reporter that allows detection of the germline leads to a 100% accurate selection of spermless males, improving the system. These males cause a striking reduction in mosquito population size when released at field-like frequencies in competition cages against wild type males. These findings demonstrate that such a genetic system could be adopted for SIT against important malaria vectors.

## Introduction

Strategies aimed at targeting insect vectors of human pathogens are central to the control of vector-borne diseases and form a vital component of the WHO malaria control and elimination program^1^. Recent successes in reducing malaria deaths have been achieved mainly by widespread implementation of two vector control strategies: long-lasting insecticide-treated nets and indoor residual spraying^2^. Together, these methods are estimated to account for over 75% of malaria cases prevented since the year 2000^3,4^, but increasing rates of insecticide resistance in mosquito populations threaten the long term efficacy of these tools^5,6^. Indeed, resistance to all four classes of insecticides currently available for malaria control has been reported^7,8^, making the development of novel vector control technologies increasingly urgent.

Targeting insect reproduction has long proven an efficacious and sustainable approach for controlling and eradicating insect pests. One such technology, Sterile Insect Technique (SIT), relies on releasing large numbers of sterile male insects*,* inducing sterility in female mates and leading to a decline in the target insect population^9,10^. For SIT to be effective, sterile males need to be highly competitive against wild type males and effectively inhibit wild female remating^11^. Traditionally, sterilization is achieved through irradiation or chemical-based sterilization methods to induce lethal DNA mutations in germ cells through oxidative stress^12^. However, these methods of sterilization also impair overall male mating competitiveness: somatic DNA, lipid, and protein oxidation synergize to impact various life history traits^13^, which combined severely reduce the male’s ability to compete for mates^14–18^.

Developing sterilization methods that specifically target fertility genes may provide an alternative avenue to produce males that are fit for mating. Multiple, more precise, transgenic sterilization systems have been developed in some mosquito vectors, including those which preserve male fertility but kill offspring in post-embryonic developmental stages^19–22^, those which express pro-apoptotic factors in the testes^23^, and those which combine male sterilization and female-killing^24^. While these systems cause transient species-specific population suppression following release, none have yet been adopted in the most important African malaria vector *Anopheles gambiae*. Fertility-reducing selfish genetic elements have been developed in this species using CRISPR/Cas technology^25,26^. These gene drive systems are very promising, although they can face rapid evolution of genetic resistance which hinders their application in the field^27^. Importantly, the self-autonomous mode of propagation of gene drives necessitates safe mechanisms for containment and release which are not currently available^28^. Malaria control would undoubtedly benefit from the development of alternative genetic sterilization systems that expand the genetic toolkit available to limit *An. gambiae* populations across Africa.

Similar to the precision-guided (pg) SIT system developed recently in *Drosophila melanogaster *and* Aedes aegypti*^24,29^*,* here we developed a safe, self-limiting and non-invasive CRISPR-based sterilization technology in *An. gambiae* that specifically disrupts a germ cell gene for SIT-based control of wild populations. Our target is *zero population growth* (*zpg)*, a gap junction innexin which plays a crucial role in early germ cell differentiation and survival^30^ and has been shown to be required for germ cell development in *Drosophila*^30,31^ and mosquitoes^32,33^. The *zpg* promoter has been demonstrated to express in a germline-specific manner^34^, and in *An. gambiae zpg* knockdown by transient RNAi results in sterile males with phenotypically atrophied testes^32^. Importantly, these males initiate typical post-mating responses in females following copulation and remain competent at mating, making *zpg* an ideal gene target for genetic sterilization. To generate sterile males, we developed a transgenic CRISPR system that achieves inducible mutation of *zpg* following a single cross of a germline-restricted Cas9-expressing line to a *zpg*-targeting gRNA-expressing line. We show that mosaic mutagenesis in the germlines of F1 males inheriting both transgenes is sufficient to achieve synchronous biallelic knockouts of *zpg* in the developing germline, ablating sperm development in 95% of males. Moreover, these males render females infertile after mating, and cause significant population suppression in competition cages against wild type males. With some adaptations, this system could be used for large-scale sterile male releases, providing a critical novel tool for self-limiting malaria vector control.

## Results

### Male Δ*zpg* mosaics fail to develop normal testes

**Figure 1:**
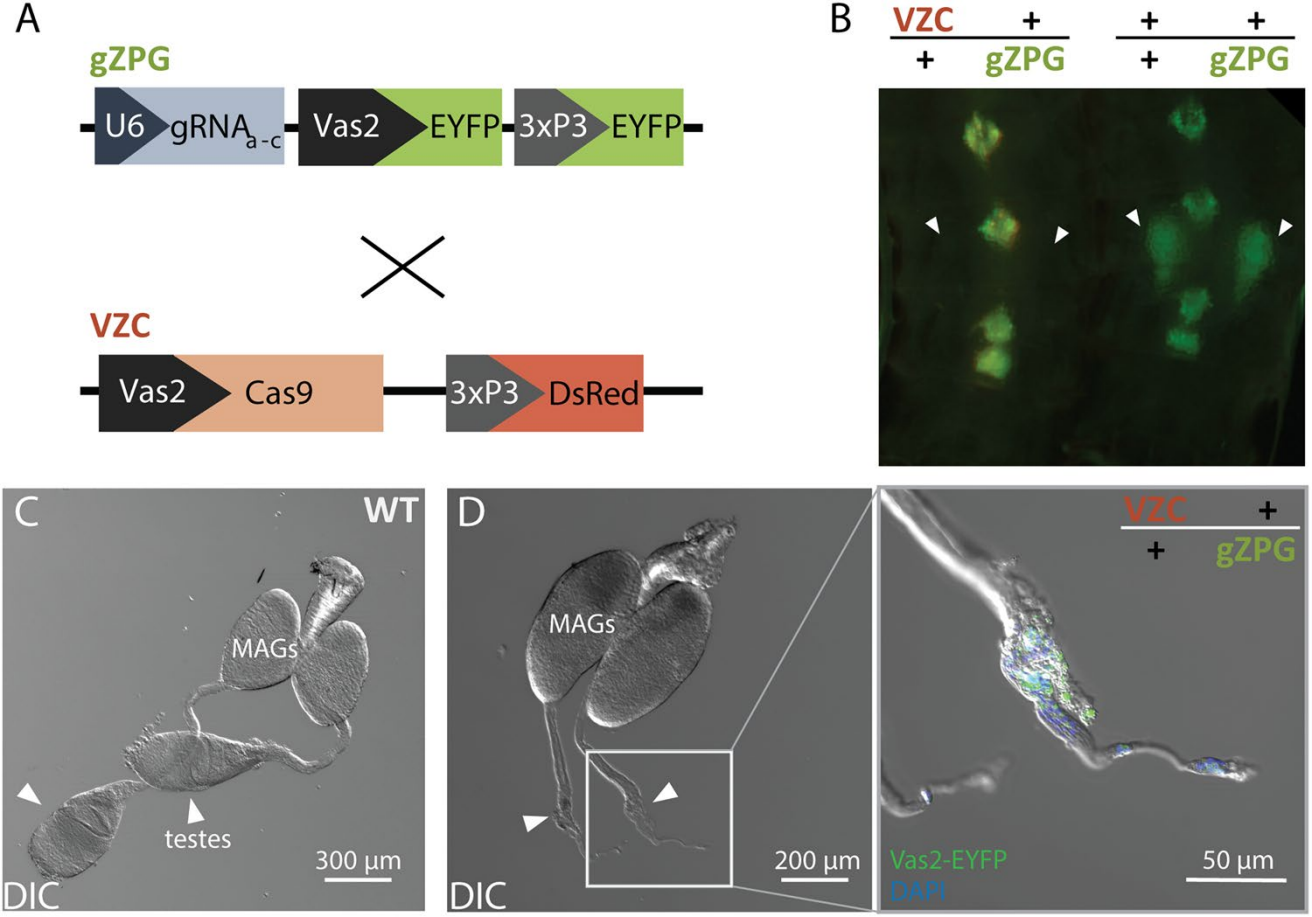
Crossing VZC and gZPG transgenic individuals generates spermless males. (**A**) A schematic representation of the VZC and gZPG constructs used to generate (VZC/+ ; gZPG/+) males. These transgenic lines were previously described^35^. In brief, VZC expresses Cas9 via the *Vas2* promoter and carries a 3xP3-DsRed marker for selection. The transgene gZPG expresses three gRNAs (gRNA_a_, gRNA_b_ and gRNA_c_) under the RNA PolIII promoter *U6*, in addition to a *Vas2*-EYFP germline marker and a 3xP3-EYFP selectable marker. Note the *Vas2*-EYFP fluorescent germline selectable marker that was used to screen for males with no clear evidence of sperm in their testes. (**B**) Fluorescent testes can be observed through the pupal cuticle alongside the 3xP3-EYFP neural marker in gZPG/+ males but not hybrid (VZC/+ ; gZPG/+) males**.** White arrows indicate the presence or absence of testes visible by fluorescence through the pupal cuticle. Image taken with fluorescence microscopy. (**C**) Wild type male reproductive tract showing male accessory glands (MAGs) and sperm-filled testes (arrowheads). Image taken with Differential Interference Contrast (DIC) microscopy. (**D**) In (VZC/+ ; gZPG/+) males, testes fail to develop (arrowheads), with minimal *Vas2*-EYFP and DAPI staining observed. Image taken with Differential Interference Contrast (DIC) microscopy (left panel) and merged with fluorescence microscopy (right panel).

To generate spermless males, we crossed males expressing guide RNAs targeting *zpg* (gZPG line) to females expressing a germline-specific Cas9 (VZC line) (Fig. 1A). (VZC/+ ; gZPG/+) offspring underwent significant mosaic mutagenesis in the germline, resulting in abnormal testes in the majority of males, but their sex ratio was unaffected (53% male, n = 622, *p* > 0.05, Fisher’s exact test). This phenotype was robustly detectable from the pupal stage by the absence of fluorescence from a *Vas2*-EYFP reporter in the seventh abdominal segment (Fig. 1B). Dissecting the reproductive tract from 126 adult males revealed atrophied testes with no visible mature sperm in 120 individuals (95.2%), in contrast to wild type controls (Fig. 1C,D). A small minority of males showed however some level of germline differentiation and sperm development, having developed a single testis (5/126, 3.96%). A single male developed both testes (0.79%). In all 126 individuals, other reproductive tissues were unaffected, with male accessory glands appearing normal.

**Figure 2:**
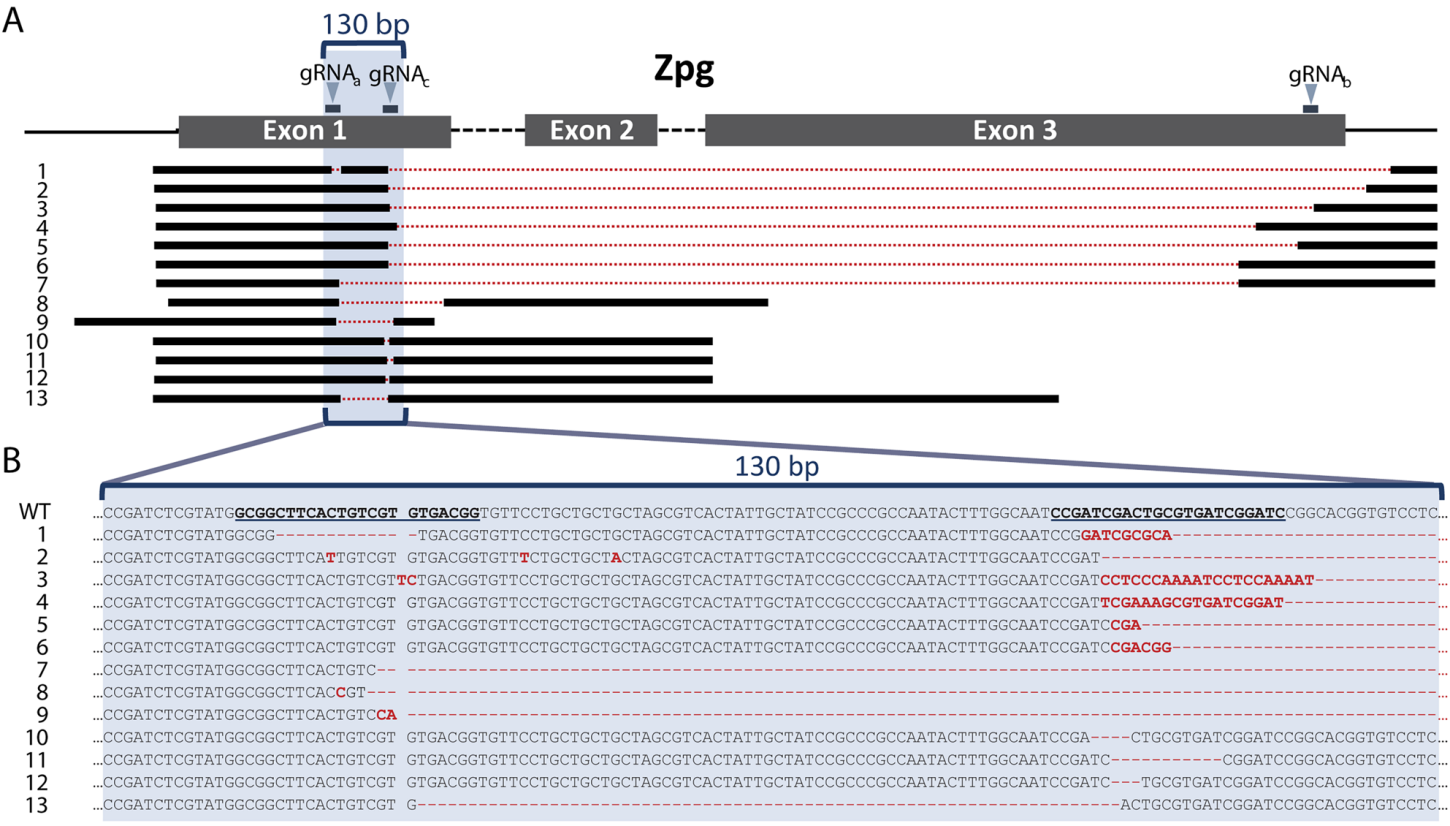
Germline CRISPR/Cas9 activity generates multiple large deletions in *zpg*. (**A**) A representative map of observed mutations summarizing large deletions in the three exons of *zpg*. Positions of the three gRNAs used in this work are shown to scale. A 130 bp sequence encompassing gRNA_a_ and gRNA_c_ target sites is shaded in blue. Sequence 13 belongs to a fertile male, while all others belong to sterile males. (**B**) Sequences of observed mutations in the region between gRNA_a_ and gRNA_c_ (underlined). Sequences 1–13 correspond to 1–13 shown above in (**A**). Inserted bases are labelled in red and deleted regions are indicated by red dotted lines.

**Figure 3:**
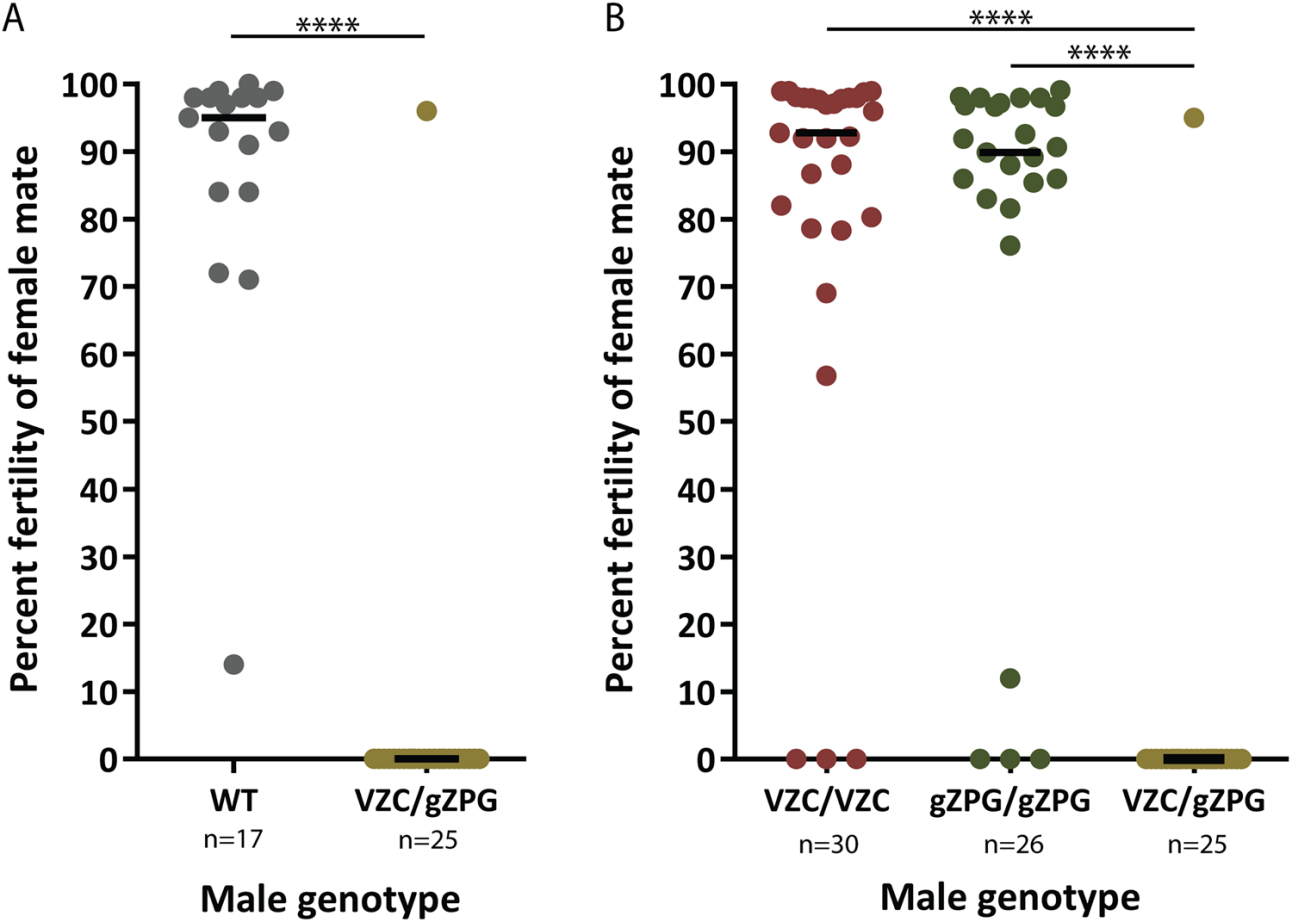
Δ*zpg* males are highly sterile. (**A**) Forced mating assays between WT female and either (VZC/+ ; gZPG/+) or WT males show most transgenic males are completely sterile (Mann Whitney, *p* < 0.0001). (**B**) Forced mating assays between WT female and individual males of the (VZC/VZC), (gZPG/gZPG), or (VZC/+; gZPG/+) genotypes show the parental transgenes have no effects on male sterility (Kruskal–Wallis, *p* < 0.0001).

We sequenced the testes or offspring of similar (VZC/+ ; gZPG/+) males and confirmed several CRISPR-induced mutations, mostly large deletions between the three gRNA target sites (Fig. 2A), and some insertions (Fig. 2B). Although this observation is qualitative, many of the large deletions observed appeared to result from mutagenesis under both gRNA_b,_ targeting the 3’ end, and gRNA_c_ at the 5’ end, with fewer initiated by gRNA_a_, suggesting differential cleavage capabilities of gRNA_c_ and gRNA_a_. Multiple mutations were observed within individual males (Fig. 2, sequences 7 & 10; 8 & 9). Among males that showed one or two testes, some sired progeny, and sequencing their testes or offspring revealed either no evidence of mutagenesis (and their sequences are therefore omitted from Fig. 2B) or an in-frame deletion; one fertile male (Fig. 2B, Sequence 13; Fig. 3A) harbored a 69 bp in-frame deletion roughly corresponding to the 4th transmembrane domain of ZPG, suggesting sperm production can be maintained even in the presence of larger deletions. In a similar way, ovarian development in females was strongly affected, greatly reducing their fecundity after blood feeding in agreement with previous observations (Fig. S1A)^35^. These data indicate that CRISPR mutagenesis of the male germline causes high levels of testis disruption but is not fully penetrant, and some fertility-maintaining mutations are possible.

### Male Δ*zpg* mosaics are highly sterile

The absence of visible sperm in most (VZC/+ ; gZPG/+) males suggested that they should be sterile, making them good candidates for use in SIT programs. To test this, we released (VZC/+ ; gZPG/+) males into a cage with an excess of wild type (+ / +) virgin females, and allowed them to mate for two nights. Females were then blood fed and allowed to lay eggs. Of the 4132 eggs laid, only 3.03% were fertile, indicating high levels of sterility in females mated to (VZC/+ ; gZPG/+) males. To determine if hatched larvae were sired by a few fully fertile males or whether each male had some level of fertility, we performed individual forced mating assays between wild type females and (VZC/+ ; gZPG/+) males or wild type male controls, and assayed for fertility. While the vast majority of females mated to wild type males showed high fertility (more than 95%), females mated to (VZC/+ ; gZPG/+) males showed complete sterility in 24/25 cases (96%) (Fig. 3A). The single female showing normal fertility levels produced a brood with an expected 50% (VZC/+): 50% (gZPG/+) transgene ratio and sequencing DNA derived from a pool of her offspring revealed a 69 bp in-frame deletion (Fig. 2B, Sequence 13). These results confirm that a minority of (VZC/+ ; gZPG/+) Δ*zpg* mosaic males maintain normal levels of fertility, likely due to failed mutagenesis or mutations that maintain fertility. Again, in a similar way, female fertility was also reduced when (VZC/+ ; gZPG/+) females were mated to WT males (Fig. S1B). Additional mating experiments using the parental (gZPG/gZPG) and (VZC/VZC) lines demonstrated that sterility is a product of *zpg* mutagenesis induced by the presence of both transgenes rather than non-specific effects of individual transgenes, as females mated to either (gZPG/gZPG) or (VZC/VZC) males had fertility levels comparable to females mated to wild types (Fig. 3B).

### Male Δ*zpg* mosaics cause population suppression in cage releases

**Figure 4:**
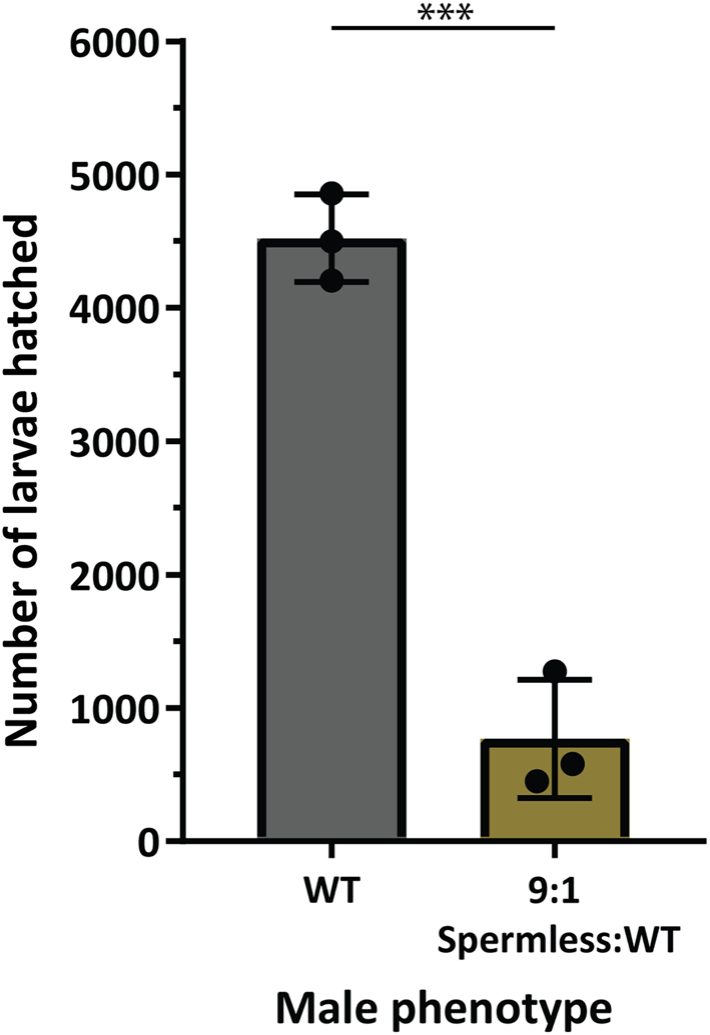
Δ*zpg* males can effectively suppress larvae numbers in competition cage experiments. 100 WT males, or a mixture of 90 (VZC/+ ; gZPG/+) males (‘Spermless’), selected for lack of *Vas2*-EYFP fluorescence, and 10 WT males, were allowed to mate with 100 WT females. After blood feeding and oviposition, the total number of hatched larvae was decreased in male competition cages compared to control cages (Unpaired two-tailed t-test, *p* < 0.001).

To be useful in SIT, genetically sterile males must be able to compete for female mates against field males. We tested whether (VZC/+ ; gZPG/+) males could suppress female fertility in competition with wild type males by simulating field releases in large cage assays. We used a 9:1 release ratio that is in line with ratios utilized in SIT strategies by introducing 90 (VZC/+ ; gZPG/+) males and 10 (+ / +) males for three nights into cages containing 100 age-matched virgin females (9:1 Spermless:WT cages). For these experiments, we only selected males that showed no testes when analyzed by fluorescence, based on expression of the *Vas2*-EYFP germline marker. As control, we set up cages where only wild type males and females were introduced (WT cages). Following blood-feeding, in three replicate experiments we observed an 83% reduction in the number of larvae hatched in experimental cages compared to control cages (Fig. 4). Microscopic analysis of larvae from the experimental cages confirmed that none had been sired by transgenic males (0 out of 2306), suggesting these males are completely sterile. Using these data, we calculated Fried’s Competitive Index^36^ and found that females were on average 4.1x (range: 2.6–7.4x) more likely to mate with wild type males (Table 1), indicating the presence of some fitness costs associated with transgenic (VZC/+ ; gZPG/+) males. In competition cages, while the egg hatch rate was lower as expected, WT females laid fewer eggs overall, suggesting many females remained unmated due to the lower fitness of transgenic males. We also observed a small decrease in wing length (a good proxy for male size, which is known to be linked to mating competitiveness^37^) in (VZC/+ ; gZPG/+) males (Fig. S2A) (Δ WT—(VZC/+ ; gZPG/+) = 46 ± 21 µm; *p* = 0.031), but not in parental VZC or gZPG lines (Fig. S2B). The mating defect stemmed from the gZPG parental line which, despite being backcrossed to WT four times, also suffered low rates of male mating to WT (20/45, 44.4% mated) or VZC (22/44, 50% mated) females after being permitted to mate ad libitum for four nights. Nevertheless, despite these fitness effects, these results demonstrate that genetically sterile males can maintain sufficient mating competitiveness to achieve significant population suppression in a competitive laboratory setting, on par with recent studies outlining pgSIT in the species^38^.

**Table 1:** Δ*zpg* males selected for lack of *Vas2*-EYFP fluorescence have reduced fitness relative to wildtype males but can still effectively suppress numbers of offspring in competition cage experiments.

	Control 100 F: 100 WT: 0 (VZC/+ ; gZPG/+)			Competition 100F: 10 WT: 90 (VZC/+ ; gZPG/+)			
Experiment	Eggs	Larvae	Hatch rate (H_N_)	Eggs	Larvae	Hatch rate (H_O_)	Fried’s Competitive Index
1	5239	4860	0.928	1412	450	0.319	0.212
2	5040	4498	0.892	2891	580	0.201	0.383
3	4562	4205	0.922	3066	1276	0.416	0.135
			Mean: 0.914			Mean: 0.312	Mean: 0.243

Numbers of larvae are plotted in Fig. 4.

## Discussion

Generating sterile male *Anopheles* has historically faced developmental hurdles. Chemo- and radio-sterilization protocols have been developed^39^, but generally cause a reduction in male competitiveness due to accumulated oxidative damage to cellular DNA, lipids and proteins^14–18, 40, 41^. Moreover, chemical sterilization raises environmental concerns due to chemical residues after mass releases^42^. GM technologies such as RIDL and pgSIT show great promise^19–21, 24^ but have yet to be adopted in *An. gambiae*. Here we outline a system for generating genetically sterilized *An. gambiae* males that could be used in SIT-like programs against this important disease vector. We show that crosses between transgenic individuals expressing Cas9 in the germline and individuals expressing gRNAs targeting *zpg* efficiently produce sterile male F1 progeny. In the vast majority of cases, F1 males have atrophied testes, show no observable sperm, and harbor numerous CRISPR-generated mutant alleles that arise by active mosaic mutagenesis during development. When not pre-screened for testicular development by fluorescence, approximately 95% of these males completely sterilize their female mates, consistent with the penetrance of the mosaic spermless phenotype. We further demonstrate that removing males showing incomplete penetrance of the spermless phenotype by screening for *Vas2*-EYFP fluorescence at the pupal stage generates male populations that are completely sterile.

Anophelines are known to mate in large swarms with highly skewed sex ratios where competition between males is fierce^43^. Competition cage assays with (VZC/+ ; gZPG/+) males show that transgenic spermless males, although less competitive, can cause significant population suppression in the presence of wild type males. Reduced mating competitiveness has often been observed with other sterilization methods. In the 1960s and 1970s chemo-sterilization was used to generate sterile males^44^ but it exhibited peripheral mutagenic effects^42^. Sterilization by radiation therefore became the dominant technique for most insects, and factors like age, stage, handling, oxygen level, ambient temperature and dose-rate were shown to be important to generate insects with sufficient competitiveness^45^. In anophelines, irradiation at the adult stage, rather than the pupal stage, produces more competitive males^39,40^, but adult fitness is maximized only when a partially-sterilizing radiation dose is used, hindering suppression effects in trials^40^. While males have similar longevity to wild type competitors^40^, they nevertheless fail to compete for females, even when released in excess of modeled recommendations^41^. We observed a decrease in (VZC/+ ; gZPG/+) male fitness associated with the gZPG transgene as many females did not lay eggs in competition cages and likely remained unmated. The mating defect is probably due to gZPG transgene position or bottleneck effects associated with the line’s creation as other transgenes in the laboratory incorporating similar components (*Vas2* and *3xP3* promoters, fluorescent proteins, or *U6*-driven gRNAs) do not have reproductive phenotypes^46,47^. While we cannot exclude that the combination of gZPG and VZC transgenes harm males in additional unanticipated ways through, for example, off-target mutagenesis^48^, the data suggest that specifically mutating *zpg* does not significantly further impair male fitness per se. This is consistent with previous studies that produced mating-competent spermless males using RNA interference against *zpg*^32^. Although obtained in limited laboratory conditions, our data show transgenic spermless males can achieve population suppression in laboratory cages. In future studies, additional reduction of the fitness costs associated with transgenesis and direct characterization of male mating competitiveness in semi-field settings will be critical to determine how this genetic sterilization system compares to traditional radiation-based sterilization techniques.

While our system shows promise for vector control, multiple steps of optimization will be required to render it functional in field settings. First, SIT strategies aim to release males that are > 99% sterile, while we observed 5% of males escaping sterilization^10^. To this end additional gRNAs could be used to boost genetic sterility but it will be important to understand the properties required for optimal DNA cleavage in the species. Others have shown that gRNAs vary in their mutagenic potential^49^, an observation qualitatively supported by our findings where gRNAc catalyzed more mutations than gRNA_a_, however this could also be due to variations in gRNA design as these gRNAs carry different scaffolds. Alternatively, additional genes important for fertility could be targeted, such as those shown in *Drosophila* to be required in the germline, including *Tudor* (AGAP008268), *β2-tubulin* (AGAP008622), or *Vasa* (AGAP00857) among many possible candidates (reviewed in^50^). Optimization of the system to increase phenotype penetrance through genetic means, and/or addition of a fluorescent sorting step to remove partially sterile males would strongly improve the chance of successful suppression. Second, our system does not allow the automatic elimination of females from the released population, an essential requirement for any male release program^10^. Combining genetic sterility with genetic sex separation systems such as those recently developed using CRISPR targeting of *femaleless*^51,52^ is therefore a necessary next step to operationalize genetic SIT for anopheline vectors.

Thirdly, given that highly sterile mosquito lines cannot breed by design, this presents a significant barrier to the large-scale production of males for vector control programs. Ideally, sterility would be suppressed during rearing stages and then triggered just prior to release. This problem has been solved in RIDL systems in *Aedes* by tetracycline-mediated repression of a lethal transgene during development, which becomes activated in offspring following release^20^. In genetic SIT, two fully fertile transgenic lines are maintained and crossed to produce infertile Δ*zpg* mosaic males and females on demand. Although more cumbersome than RIDL as two lines must be reared, this system facilitates mass rearing at scales sufficient for release, with VZC females having slightly reduced fecundity but similar fertility to WT females (Fig. S3). While this system requires significant optimization before it can be utilized in field settings, our work provides a valuable proof-of-principle that transgenic sterilization can enable SIT programs aimed at suppressing *Anopheles* populations.

Finally, it is important to note that, beyond its potential application for vector control, our system can be used to explore a variety of biological questions. Firstly, the role of sperm in regulating aspects of the female post-mating response is still largely unexplored. *An. gambiae* females display two major responses after copulation: the stimulation of oviposition following blood-feeding, and the induction of refractoriness to further mating. Both are initiated following sexual transfer of factors, including a male steroid hormone^53^ from the male to the female atrium during copulation^53–55^. Although a previous study showed that sperm is not involved in triggering these female responses^32^, the use of transgenic spermless males may identify more subtle effects linked to sperm transfer and storage. Indeed, in *Drosophila,* sperm is needed to extend the mating refractoriness period up to a week by signaling through the slow release of male-transferred sex peptides bound to sperm tails^56–58^. The Δ*zpg* mosaic males generated here could therefore be used to study the effect of sperm on similar post-mating responses in female mosquitoes, opening an intriguing avenue of research of significant importance for mosquito reproductive biology.

## Methods

### Husbandry conditions

*Anopheles gambiae* (G3 strain) were reared in cages (17.5 or 24.5 cm^3^, Bugdorm) and larval pans (32.4 cm (L) × 26.5 cm (W) by 6.4 cm (D), Cambro 22CW148) under a 12 h light: 12 h dark cycle in a facility maintained at 27 °C. To maintain 70–80% humidity, cups of water covered in paper towels were inverted atop each cage. Adults were maintained on a 10% glucose solution ad libitum*,* fed via a rolled up filter paper within a 25 mL Erlenmeyer flask. Females were fed on purchased human blood (Research Blood Components, Boston, MA) and males and females were sex separated as pupae to ensure virginity. Stock wild type G3 (the ancestral line for VZC and gZPG) was PCR amplified to verify *Anopheles gambiae* status^59^.

### Generation of transgenic mosquito lines

#### gRNA design

Design of gRNAs for these transgenic lines was previously reported in^35^. Briefly, the *zpg* locus (AGAP006241) was targeted by three gRNAs chosen to maximize the probability of mutagenesis early in the coding sequence, with the additional aim of achieving large deletions. Two gRNA candidates were chosen, gRNA_a_ and gRNA_c_, targeting the sequences (5’ GCGGCTTCACTGTCGTGTGACGG 3’) and (5’ CCGATCGACTGCGTGATCGGATC 3’) within Exon 1 located 71 bp and 150 bp from the start codon respectively. They were further chosen for their localization over semi-unique restriction enzyme sites *Ale*I and *Pvu*I respectively to enable PCR-based identification of mutants, as previously described in^60^. gRNA_b_ (5’ CCAAGTGTTTGCATTCCTGGCGG 3’) was designed to target the 3’UTR sequence to facilitate generation of large deletions. gRNA_a_ was designed to carry a variant scaffold sequence of (5’ GTTTTAGAGCTATGCTGAAAAGCATAGCAAGTTAAAATAAGGCAGTGATTTTTAATCCAGTCCGTACACAACTTGAAAAAGTGCGCACCGATTCGGTGC 3’), gRNA_b_ was designed to carry a variant scaffold sequence of (5’ GTTCCAGAGCTATGGAAACATAGCAAGTTGGAATAAGGCTAGTCCGAATTCAACTTGAAAAAGTGGCACCGAGTCGGTGCATTTTTT 3’) and gRNA_c_ was designed to carry the standard *S. pyogenes* scaffold of (5' GTTTTAGAGCTAGAAATAGCAAGTTAAAATAAGGCTAGTCCGTTATCAACTTGAAAAAGTGGCACCGAGTCGGTGCTTTTTT 3’). All gRNAs were expressed under the *An. gambiae* RNA Pol III *U6* promoter.

#### Plasmid construction

Details of plasmid construction for these transgenes have been reported previously^35^. In brief, plasmids were constructed using standard molecular biological techniques and Golden Gate cloning^61,62^ into the multiple cloning sites of *An. gambiae* transgenesis plasmids pDSAY (attB, 3xP3-EYFP fluorescence marker) and pDSAR (attB, 3xP3-DsRed fluorescence marker)^63^. SpCas9 (Addgene plasmid PX165) was placed under the control of a 2.3 kb *Vasa2* promoter (*Vas2*) and an SV40 terminator and inserted into pDSAR. gRNAs under the *U6* promoter and a *Vas2*-EYFP-SV40 cassette were inserted into pDSAY. Complete plasmids were sequence verified by Psomagen Sequencing services (Rockville, MD, USA).

#### Transgenesis

Transgenesis procedures were carried out as previously described^35,64, 65^ with constructs (350 ng/µl) co-injected with ΦC31-integrase expressing helper plasmid (80 ng/µl). gZPG and VZC were injected into *An. gambiae* X13 and X1 docking lines, respectively^63^. Injected survivors were reared to adulthood and outcrossed in bulk to large cages of wild type *An. gambiae* G3 virgin adults (n > 1000) of the opposite sex. New transformants were identified and isolated as newly hatched larvae in the subsequent F1 generation by fluorescence. F1 transformants were outcrossed to wild type G3 to introduce genetic diversity before intercrossing to establish homozygous lines by fluorescence intensity of the 3xP3 marker.

### Generation of spermless (VZC/+ ; gZPG/+) males

To generate spermless males in bulk, (gZPG/gZPG) males were crossed to virgin (VZC/VZC) females in cages. Maternal deposition of Cas9 from VZC females facilitated increased mutagenic loads in the developing embryos leading to more penetrant mosaic phenotypes. Male pupae/adults were manually sex sorted from females under a microscope using a paintbrush. For forced mating experiments, spermless males were sex separated as pupae to guarantee virginity and their genotype was confirmed by dual (*3xP3*-EYFP; *3xP3*-DsRed) fluorescence. For caged competition experiments, male pupae were additionally screened for the absence of *Vas2*-EYFP from testicular tissues to remove males with an incompletely penetrant phenotype.

### Microscopy

Imaging of transgenic larvae and ventral pupal tails was carried out under a Leica M80 fluorescence dissecting microscope following immobilization on ice and positioning by paintbrush. Imaging of microscopic testes structure was carried out on a Zeiss Inverted Observer Z1 microscope following dissection in 1 × PBS, and mounting in VECTASHIELD^®^ Mounting Medium with DAPI within 1 h post-dissection. Tissues were dissected from 5-day-old virgin males.

### Mutation analysis

Male (VZC/+ ; gZPG/+) mutant testes or surviving unsexed larvae were analysed for mutations by PCR and sequenced. DNA extraction was carried out using the Qiagen DNeasy Blood & Tissue Kit, and PCR was carried out using a variety of primers flanking the *zpg* locus. Multiple primer pairs were used to capture large deletions and enable amplification over polymorphic regions. The forward primers (5’ CGTTTTCTTCACTCTCGGCACG 3’), (5’ GCAGCTTCTGGTAGTCGATGTCG 3’), and (5’ CCATTCGTTTGTTGCTGAAAGC 3’), and reverse primers (5’ GACCAGAAGCCGGAAAAGATC 3’), (5’ GAGGAACGCGGGTTTTTTTG 3’), and (5’ GTGAAATGTTTGGGCCCGATC 3’) were used in combinations to generate PCR products ranging from 700 bp to 5 kb. Occasionally, PCRs were not successful, likely due to limited DNA extracted from atrophied mutant testes, or the absence of any primer binding sites. Individual mutant alleles were sequenced essentially as previously described^60^. PCR products were cloned into the CloneJet PCR Cloning Kit (ThermoFisher Scientific) to isolate PCR products corresponding to individual alleles, and plated on ampicillin (100 µg/mL) LB media plates. Individual colonies were either picked, cultured in liquid media, extracted (SpinSmart Plasmid Miniprep DNA Purification kit, Denville Scientific) and sequenced using the universal pJET2.1F or pJET2.1R primers (Psomagen USA), or the entire agar plate was sent for direct colony sequencing (Psomagen USA). Resulting sequencing reads were aligned to an annotated Snapgene 3.2.1 file of the *zpg* gene sequence.

### Infertility mating assays

#### Bulk mating

30 (VZC/+ ; gZPG/+) males were sexed as pupae and allowed to eclose into a 25 cm × 25 cm BugDorm cage (MegaView Science co, Taiwan). Four failed to eclose, leaving 26 surviving males for the experiment. Female pupae of the wild-type strain G3 were sexed on the same day and allowed to eclose in a separate cage. The absence of contaminating G3 males was confirmed the next morning, and 176 females were mouth-aspirated into the cage containing the (VZC/+ ; gZPG/+) males. Female mosquitoes were allowed to mate for 4 nights, and were blood fed on day 5 until significant diuresis was observed. An oviposition site consisting of a Whatman^®^ filter paper cone (90 mm, Grade 2, Sigma-Aldrich) within a urinalysis cup containing 80 ml deionised water was placed in the cage on day 7. The oviposition cup was removed on day 8, and larvae were counted and scored for transgene presence on day 9. Eggs and late-hatching larvae (none observed) were counted on day 11 and 12.

#### Individual forced-mating assays

5 days-post eclosion, virgin males of respective genotypes and blood-fed virgin wild type G3 females were force-mated to guarantee paternity (method available at https://www.beiresources.org/MR4Home.aspx). In brief, males were anesthetized on ice, decapitated and mounted on the head of a pin while females were anesthetized on a nitric oxide pad (Inject + Matic). Copulatory behaviors were recapitulated by bringing the claspers of the male towards the gonotreme of the female. Male carcasses were saved for subsequent mutation analysis. Successful mating was confirmed by autofluorescence of the mating plug in the female atrium, detectable through the female cuticle under a fluorescent microscope using a GFP filter set (previously demonstrated in^54^), and females were isolated to oviposit within individual paper cups lined with filter paper and filled with 1 cm deionised water. The number of eggs laid and larvae hatched were counted from each female’s brood, and larvae screened for transgene fluorescence to determine paternity. Escapee larvae sired by genetically sterilized (VZC/+ ; gZPG/+) fathers were collected for subsequent sequence analysis.

#### Cage competition assays

(VZC/+ ; gZPG/+) *Vas2*-EYFP-negative males and wild-type G3 males and females eclosed into separate cages, and adults were mixed at 3 days old to allow mating in Bugdorm 4M2222 cages (24.5 cm × 24.5 cm × 24.5 cm). Control cages contained 100 G3 males; 100 age-matched females, and competition cages contained 90 (VZC/ + ; gZPG/+) *Vas2*-EYFP-negative males and 10 G3 males; 100 G3 females. At 6 days old, females were offered a blood meal for 20 min and males were removed. An oviposition site was provided to females at 8 days old and was removed when 10 days old, and eggs and larvae were counted on days 10 and 11, with larvae scored for genotype (and therefore paternity) by fluorescence. Fried’s Competition Index^36^ was calculated as ((H_N_ − H_O_)/(H_O_ − H_S_))*(N/S), where H_N_ (normal) and H_S_ (sterile) indicate hatch rates of eggs laid by females mated to either normal or sterile males, H_O_ indicates the observed hatch rate of eggs laid by females in the competition assay, and N/S is the ratio of numbers of normal to sterile males (10/90 in our experimental design). A value either above 1 or below 1 indicates females are more likely to mate with (VZC/+ ; gZPG/+) *Vas2*-EYFP-negative males or wild-type G3 males, respectively.

### Wing length measurement

Wings were dissected and imaged under brightfield illumination at 2.5X on a Leica M80 fluorescence dissection microscope fitted with a Leica DFC310 FX camera. Damaged wings were excluded. Images were scaled using a graticule and FIJI software^66^. Length measurements in millimeters were taken from the proximal wing notch to the point where the third wing vein reaches the distal tip of the wing (excluding wing scales).

### Supplementary Information

Supplementary Figures.

## Data Availability

The datasets generated and analyzed in the current study are either included in this published article (Figs. 1 and 2) or are available in the Harvard Dataverse repository under the identifier https://doi.org/10.7910/DVN/DVYGST (Figs. 3, 4 and S1–3 and Table 1).
