# Supplementary material for: CRISPR-mediated germline mutagenesis for genetic sterilization of Anopheles gambiae males
Source: Sci Rep. 2024 Feb 19;14:4057. doi: 10.1038/s41598-024-54498-8 (PMC10876656; doi:10.1038/s41598-024-54498-8)
Supplement: Supplementary file 1 — Supplementary Figures. [file 41598_2024_54498_MOESM1_ESM.pdf]

Supplementary Information for

CRISPR-mediated germline mutagenesis for genetic sterilization of *Anopheles gambiae* males

Andrea L. Smidler[1,2,3], Eryney Marrogi[1], Jamie Kauffman[1], Douglas G. Paton[1,4], Kathleen A. Westervelt[1], George M. Church[2], Kevin M. Esvelt[5], W. Robert Shaw[1,6*], Flaminia Catteruccia[1,6*]

*Email: wrshaw@hsph.harvard.edu, fcatter@hsph.harvard.edu

This PDF file includes:

Figures S1 to S3

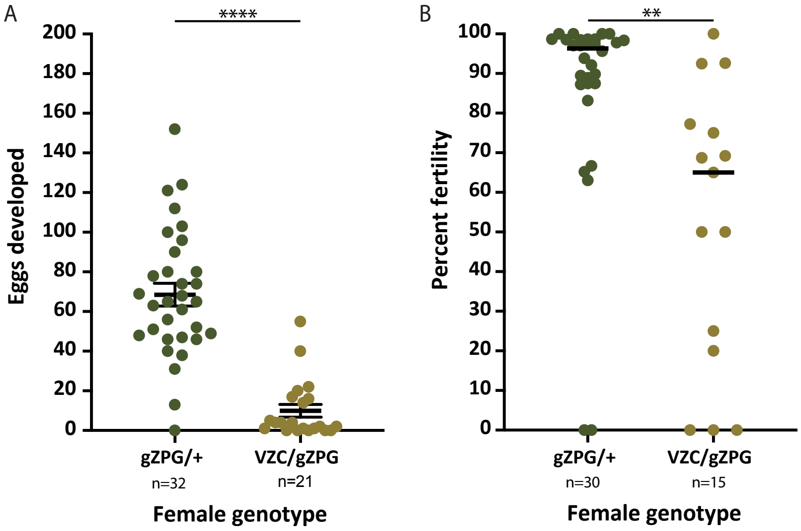

Figure S1. **Δ*zpg* females develop few eggs and have high rates of infertility.** (gZPG/+) or (VZC/+; gZPG/+) females were mated to WT males, blood fed and allowed to oviposit in individual egg cups. Females were dissected to score mating (by checking for sperm in the spermatheca), the presence of ovarian follicles ((VZC/+; gZPG/+) females with no ovaries were excluded), and the numbers of retained eggs. (VZC/+; gZPG/+) females **(A)** develop far fewer eggs (Kruskal-Wallis, $p < 0.0001$) and **(B)** have higher rates of infertility (Kruskal-Wallis, $p < 0.01$) than (gZPG/+) controls.

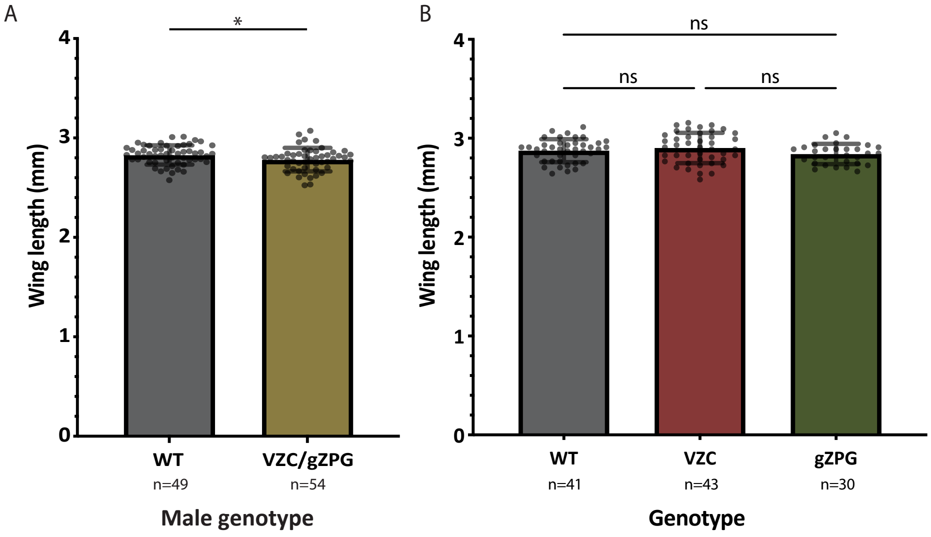

Figure S2. **Wing lengths of transgenic lines used in this study. A)** Following the mating competition assays, males were removed, sorted by genotype by fluorescence, and their wings measured using FIJI software. Mean wing length was slightly smaller in (VZC/+; gZPG/+) mutant males (Δ WT – (VZC/+; gZPG/+) = 46 ± 21 μm; Unpaired two-tailed t-test, $p = 0.031$). **B)** Mean lengths were not significantly different between parental VZC and gZPG transgenic lines and WT mosquitoes (ANOVA, $p > 0.05$).

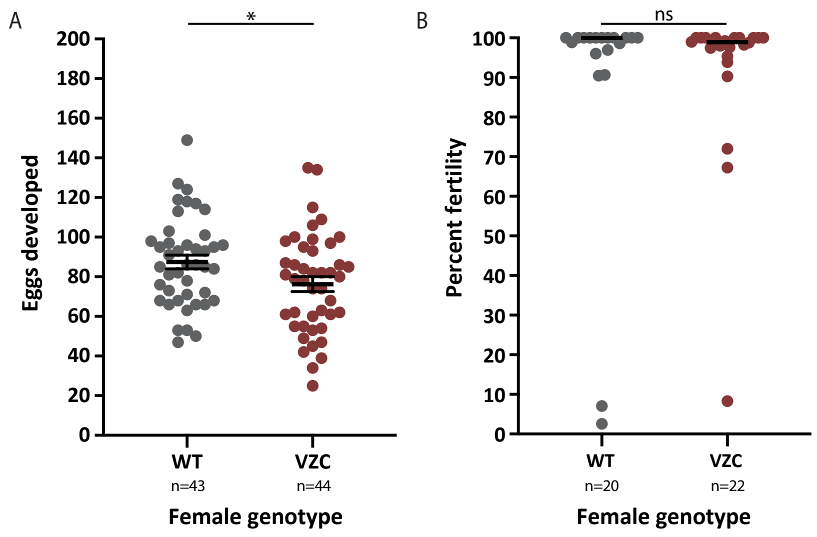

Figure S3. **VZC females are highly fecund and fertile when mated to gZPG males.** WT or VZC females were mated to gZPG males, blood fed and allowed to oviposit in individual egg cups. Females were dissected to score mating (by checking for sperm in the spermatheca), and the numbers of retained eggs. VZC females **(A)** develop slightly fewer eggs (Unpaired two-tailed t-test, $p < 0.05$) but **(B)** have similar rates of fertility (Mann-Whitney, $p > 0.05$) as compared to WT controls.
